# The Alcoholic Appetite

**Published:** 1882-06

**Authors:** Roger Marvin Griswold

**Affiliations:** North Manchester, Conn.


ARTICLE II.

### THE ALCOHOLIC APPETITE.

BY ROGER MARVIN GRISWOLD, M.D., NORTH MANCHESTER, CONN.

Men get drunk for various reasons, but the condition of drunkenness, even if frequently repeated, does not necessarily make a man a drunkard. From a legitimate point of view, he deserves this name only when the appetite for liquor drives him to take it as hunger impels him to take food.

Some of the conditions under which men drink have been ably summed up by Dr. Bird, late Surgeon-General in the British India Army, as follows :

"One man drinks to drown his sorrow, another is led into it by his convivial habits. A third drinks because he is weak-minded and is shamed into it by his stronger-minded companions ; a fourth drinks because the traditions of his trade demand that he should do so." To these I add a fifth, who drinks because an inherent quality in his nature, which he has not sufficient will-power to resist, urges him to it.

By changing the circumstances and surroundings of the first four classes mentioned, we may do much to convert them into sober men and good citizens—men who can abstain or indulge, as their reason dictates. But what shall we do for the last, who has in him that inherent appetite which is as much a part of his nature as the craving for food when hungry, or for water when thirsty ?

I have said that a "man is a drunkard who has an inherent appetite which he *has not sufficient will-power to resist ;*" but I go farther than that, and add that that man who has this appetite, and who is constantly tempted to indulge it, even though by the exercise of his will he has been heretofore, and is still a sober man, is nevertheless a drunkard.

A man may be a consumptive, but, knowing his tendency, by the exercise of extreme care he holds the disease in check, and lives to a good old age, to die of some other malady ; so this man, this latent drunkard, by the exercise of a strong will, knowing that a little indulgence will carry the appetite beyond his control, protects himself from its further development by being a total abstainer.

For the easier consideration of the subject, drunkards may be divided into three classes : the temporary, the periodic, and the chronic. The temporary, so far as I know, always appears in connection with some disease of the body (and may be considered as a symptom), or in connection with some temporary mental disturbance. It is of comparatively frequent occurrence in the recoveries from certain kinds of fevers, in tapeworm, in dyspepsia, in diseases of the brain resulting from injuries, in malarial disorders, in periods of long-continued mental depression, and often in pregnancy attended with severe nausea.

In the periodic form the attacks usually come on at stated intervals, but the duration of the attacks, and the intervals of time between them, vary in different individuals. When the attack does come on, the craving is almost irresistible, and nothing but the strongest determination, or compulsatory detention, will prevent the gratification of the appetite. So strong is the desire for stimulants, that a patient once said to me : "When the turns come on, I would walk through hell fire if I could get brandy on the other side."

Dr. J. E. Lumer relates the case of a man who, during four weeks, secretly drank the alcohol from six jars containing morbid specimens. A recent author says upon this point : "The person affected is entirely beyond self-control, and he yields to the influence of the all-absorbing appetite without even a shadow of resistance to gratify it ; every other consideration is sacrificed. He feels nothing but the insatiate desire, he thinks of nothing but the means of gratifying it. His mental powers are overwhelmed in the all-absorbing demand."

The chronic form of drunkenness is that with which we are most familiar. It is seen everywhere : under the burning suns of the equator, and in the ice-bound regions of the north ; beneath the sunny skies of Italy, and in the fog-enveloped British Isles ; where the waves of the Indian Ocean lap the shores of Hindoostan, and among the many green islands of the calm Pacific ; in the tiger-haunted jungles of Africa, and in the ice-land of the Esquimaux ; amid the barbarism of Central Asia and Central South America, and in the centres of civilized Europe, from the Atlantic to the Pacific, from the Red River of the North to the Gulf of Mexico in our own fair land, it stalks about our streets and public places like the great plague, and is the chief presiding skeleton in many homes.

Its chief symptom is daily drunkenness, more or less complete.

Dr. Robert Bird, in an essay upon "Drink-Craving" says of this form of drunkenness : "When men are afflicted with this condition, abandoning all other occupations, getting drunk becomes the chief business of their lives. The vision of alcohol fills their thoughts and imaginations at all times and in all places, shutting them out by an impassable barrier from all that is respectable and human. All they think and do has reference to this overpowering appetite that has enslaved them."

As a consequence of this constant and habitual drinking, all the tissues of the body deteriorate, the appetite for food gives place to the appetite for alcohol, the nervous vitality is more and more impaired, the natural checks, self-respect, honor, love of family, grow weaker, until in time the despotic appetite compels mind and body to abandon all else, and the victim devotes his chief and almost entire attention to procuring means for satisfying this insatiate craving.

This, more than any other form of drunkenness, is inherited, and when it is so, is very quickly developed, and most rapidly overcomes a man's self-control. Dr. Crothers, in speaking of hereditary influence in reference to this form of drunkenness, says: "Statistics indicate that it always appears in the generations following, either originally, or in some of its allied forms of disease, of which mental aberration, melancholia, idiocy, chorea, epilepsy, paralysis, apoplexy, dementia, and other nervous diseases, are most common."

While the causes which lead to chronic drunkenness are many, I think the statistics of asylums and the observation of private practitioners will confirm me in the statement, that to heredity is due a large majority of all the cases of chronic drink-craving. Other associate causes are occupation, mental construction, hygienic conditions, climate, race, and age, and most prominent among these are hygienic conditions.

Defective sanitary surroundings are certainly a very fruitful cause of habitual drunkenness. Of two men of similar temperament and constitution, he who is well fed, well clothed, and well sheltered, is certain, in a vast majority of cases, to be more temperate than he who is insufficiently or poorly fed, poorly clothed, and sheltered in uncomfortable apartments.

"A slatternly wife will make a drunken husband."

Improperly cooked meals, untidy clothes, filthy smells, disorder about the house, squalid children, and the sour face and sharp tongue of the woman who usually reigns in such a home, detract very materially from the comfort of the husband, and for the home comforts which he finds lacking, he seeks compensation in the warmth and convivial companionship connected with the dram-shop. Occasional exposure to physical hardship is seldom detrimental to health, but rather acts as a functional stimulant; but continued exposure is generally the beginning of disease, and the beginning of disease from these causes is commonly the beginning of drinking habits.

As a remedy against physical discomforts, men turn to drink as readily as they turn to fire. In damp and badly drained localities, in poorly ventilated and overcrowded tenement-houses and shops, among those who are insufficiently fed, or fed on food of an inferior quality, drunkards abound. It may be argued that drinking habits have induced the condition which subjects a man to these surroundings, but I believe rather that it may be broadly stated that nearly all underfed and poorly sheltered people turn to drink in consequence of the morbid appetite created by the physical discomforts by which they are surrounded.

That the food of a people has a direct influence upon their drinking habits I think there is but little doubt. The Scotch working people live largely upon oatmeal; the chief staple of diet among the Irish is potatoes; among the Russians rye bread. All these people are much subject to dyspepsias, due to a great extent to their farinaceous diet; and as a result of the derangement of their digestive functions they turn to drink. I have found it to be a very common occurrence that patients suffering from dyspepsia developed an appetite for stimulants, which disappeared when the digestive functions were restored to a normal condition.

Climate and race have undoubtedly much to do with the appetite for drink, and upon this point I cannot do better than quote from Dr. Bird. He says: "As differences in the social systems and in the religions which at present prevail on the globe are in some measure to be attributed to climate, so I think is the difference in the alcoholic appetite as manifest in different nations. If we examine the different substances used by different nations to gratify their love for stimulants, we find that in and about the tropics opium, Indian hemp, tobacco, and betel-nut are most used. In the northern hemisphere, as we go toward the pole, we find that light wines, strong wines, and spirits, in the order in which I have named them, are most used, and in the Arctic regions alcoholic drinks are abandoned for meats, fat, and oils. Tea, coffee, and tobacco are stimulants which are relished, and in almost universal use, all over the globe. With respect to race, it is acknowledged that it has much to do with moulding the character and developing the manners and customs of a nation, and it is not unreasonable to conclude that race has much to do as a cause affecting a nation in its relation to drink; and while attempting to offer no satisfactory explanation for that which I believe to be a fact, yet I broadly affirm that those nations of modern times which have manifested a continued impatience of despotism, and a progressive affection for social and religious liberties, are more given to strong drink than nations of an opposite tendency."

*As a nation approaches toward the highest point of social and religious liberty, just in proportion does the love for and use of strong drink appear to increase among its people.*

Mental construction has much to do with a man's drinking habits. A fool is seldom a drunkard. A close, penurious, selfish man seldom drinks. A morose, melancholy, distrustful man, thinking only of himself and his own affairs, caring only for his own interests, and unmindful of the interests of those around him, seldom acquires the habit.

On the other hand, those qualities which make men popular with their fellow-men are causes which lead men within the influence of drink, and are undoubtedly causes tending to it. If a man is a good singer, a good speaker, a good story-teller, open-hearted, open-handed, generous, and jovial—in fact, if he possess those qualities which entitle him to be called "a good fellow," he will often find himself in the position where he is subjected to the temptation to drink. Such men are generally easily influenced, and easily acquire the habit and the taste for stimulants; and the associations among which this class of men are so often thrown has undoubtedly much to do with the habit of drink which so many of them acquire.

It is a prevalent belief that many men take to drink because they are unhappy; that they try to drown their sorrow in the oblivion of drunkenness, or forget their troubles in the exhilaration of the wine-cup. If the trouble depends upon physical discomfort, this belief may be well founded; but mental unhappiness, although it may be a case of temporary drunkenness, I believe makes few chronic drunkards. As the cause of his mental trouble is removed or forgotten, he returns to his daily routine of business, and no longer desires his stimulants.

We will now notice the most important factor in the production of inebriety. The subject of hereditary inebriety has been brought into view of the general public within a comparatively few years, although recognized as an important factor in the production of drunkenness by a certain number of the medical profession.

Like many other evils that follow one generation after another, the direct outgrowth of some vicious habit in those preceding us, it has been regarded, and is still by many regarded, simply *as a habit,* having its origin in the person indulging it, while in reality he is no more to be blamed because he has an inherent appetite for alcohol, than is his neighbor who has an inherited light complexion, an inherited gout, or an inherited consumptive tendency. It is a safe rule to lay down that when either a man's father or mother, and in most cases when either of his grandparents have been for any length of time addicted to the habitual use of liquor previous to that man's birth, he will almost to a certainty have in him an inherent appetite for alcohol. Favoring circumstances may have combined to prevent the appetite from developing itself, but place that man in the way of temptation, and ninety times in a hundred he falls, and finds that appetite awakened into an activity that soon and easily becomes overpowering. It is needless for me to attempt to prove that this appetite is transmitted.

In the transmission of disease from parent to child, it is never the *disease itself* which is transmitted, but a *tendency to* the disease. Consumption, scrofula, etc. are seldom or never manifest in the child at birth, but there exists the *inherited predisposition to the disease,* out of which, sooner or later, the disease is developed. A person, for instance, with this hereditary tendency may live thirty years, during that time enjoying all the ordinary comforts of life, and exposed to no very severe hardships. But now he may be suddenly exposed to an unusual amount of hardship and privation, and he develops a consumption. Another man, under the same circumstances, shows no signs of consumption, but develops a rheumatism, while a third retains his usual health. Now this third man suffered the same hardships and exposures as the other two. He was no better qualified *apparently,* perhaps not so well, to withstand them as the others, but his parents were healthy before him, transmitting to him no disease germs to lurk in his system, awaiting a favorable opportunity for development.

A similar analogy will hold good in relation to hereditary inebriety. A man whose parents drank is removed from their influence at an early age, perhaps by their death. He reaches the age of twenty-five without knowing the taste of liquor, and without knowing that his parents had ever been in the habit of drinking. He is at this age exposed to influences that lead him to drink, and he suddenly finds himself in the hands of a giant whose grip is a hard one to unloose ; while another man of the same age, under the same surrounding circumstances, may continue to drink or let it alone, as pleases him.

Combe in his work upon "The Constitution of Man" relates the following circumstance as illustrating the transmission, from parent to child, of the alcoholic appetite: "A friend told me that in his younger days he lived in a country where the gentlemen were much addicted to hard drinking, and that he frequently took a part in their revels. Several of his sons, born at this time, although morally educated, were strongly addicted to inebriety, whereas the children born after his removal to a large town and the forming of more correct habits, were not the victims of this propensity."

This, and many other instances scattered through books and in the experience of all of us, prove that the appetite for alcoholic drinks can be transmitted from parents to children. To such an extent is this true, that the children of a drunken parent, and even the grandchildren of a drunken grandparent, require to be carefully watched and educated to prevent them from falling into the habit of drunkenness. A drunken father may not only beget a drunken son, but a drunken grandparent may leaven whole families with the dire affliction of drink-craving.

The following instance, quoted from the work before alluded to, illustrates this law:

"A gentleman farmer, living in the northern part of England, was much given to strong drink. He was fond of good living and good company. After his marriage he became more intemperate than before, and at last died a confirmed sot, from paralysis and kidney disease. He left six children, three sons and three daughters, who all in time became drunkards; but the eldest son, born when the habits of his father were not so intemperate, was less addicted to it than the others. The second son, while studying law, fell into every kind of debauchery, finally came to America, and died a sot in a hospital in New York. The third son was brought up for the ministry, and gave much promise of ability and moral worth, but he also fell into dissipated ways, and often went drunk into the pulpit. Expelled from his ministerial office, he lived a short life of poverty and drunkenness, and died in a workhouse. The eldest daughter tapped a barrel of rum in her husband's absence, and was found by him dead under it. The second daughter died in a fit of delirium tremens. The third daughter was not so violent a drunkard as her sisters, but nevertheless got drunk whenever she had an opportunity. The eldest son succeeded to his father's property, and, while he drank more moderately than his brothers, was still spoken of as intemperate. He died a violent death, leaving two sons and three daughters. His eldest son, after a short life of dissipation, died a drunkard. The second son committed a murder under the influence of drink. The eldest daughter had a delicate constitution, and died in giving birth to her first child. The second daughter died of consumption. The third daughter was confined in an insane asylum soon after her marriage.''

It is not too much to say that all this ruinous train of circumstances arose from the condition of drunkenness in one man. It not unfrequently happens that generally temperate parents have an intemperate or an idiotic child. This may result from one or both parents having been under the influence of drink when the child was begotten.

Carpenter mentions a case in which a child begotten when both parents were partially intoxicated (but who were generally temperate) was completely idiotic.

"A child, begotten the night its mother had returned from a ball, when she had indulged in considerable wine, early manifested a strong love for drink, and died a drunkard, although none of her other children were addicted to its use.''

If we consider the nature of this appetite, we shall find that *to a certain extent* it is a natural one. An appetite for a stimulant of some kind has been natural to all men, in all countries and ages. Opium, Indian hemp, tobacco, betel-nut, tea, coffee, and oil are but different articles used by different people as substitutes for alcohol. The craving for alcohol and the condition of habitual drunkenness are but abnormal and excessive developments of this desire for stimulants.

Its manifestation was as apparent in ancient times as now. There seems to be a certain instinct which has led people, at all times and in all conditions, to seek out and have recourse to stimulants. It has led and always will lead men to the discovery and manufacture of something which shall gratify the appetite for it, as instinctively as it has led them to the means of preparing fire. It is an appetite which gains size and force from its indulgence, and depends for its full development on the habitual and daily use of intoxicating stimulants. An English author correctly expresses my mind when he says: "I believe that all men can, with more or less difficulty, be made drunkards, for all have within them the natural capability of becoming so.'' Moderately indulged, it might be a sanitary and a social blessing; but the trouble is, most men *cannot* indulge in it moderately, and habitually and immoderately indulged in it becomes a curse to its victim and a social pest.

" Originally given us for wise and good purposes, it has become, by the excesses of ourselves and our progenitors, an agent to fill our prisons and our hospitals," an agent to fill our homes with sorrow and our hearts with sadness.

We can look back over the ages of the past and see the broad swaths of ruin it has cut in the ranks of humanity. Sacred Scripture tells us that Noah planted a vineyard and got then drunk.

From what remains to us of the literature of the ancient Egyptians we see that drunkenness was common in their day. The Romans and Greeks made and consumed a great quantity and variety of wines and liquors ; and Gibbon tells us that " the chief industry of the Tartars seemed to consist in the manufacture from mare's milk of a fermented liquor, possessing very great powers cf intoxication."

Mohammedans, in spite of the commands of their prophet to the contrary, drink wine freely, and in Chinese literature we find mention of the fact that wine was in use in that country more than 2000 years before the time of Christ.

The discoverers of this country found that its inhabitants took easily and naturally to " fire-water," and the stimulant effects of tobacco were already known to them ; and it seems to me from this, that the relish for alcohol at once displayed by them is one of the surest proofs that the appetite is a natural one to all men.

Salvatori* says that this natural appetite for stimulants, morbidly developed, resides in the abdominal nerves. He may be right, and he may not. We feel thirst and hunger in the throat and stomach, but we know that the seat of these appetites is in the brain. " This stimulant appetite, making itself felt in the stomach, resides in the cranium, among the other faculties, of which we have named propensities and instincts."†

It is thus that a man may suffer from temporary or chronic drunkenness as a symptom of bodily or mental disease. When a man has acquired an appetite for drink, and indulges it, we say he has a bad habit ; but " to say that a man has a bad habit is equivalent to saying that his organization has become imperfect, viciously or otherwise as the case may be, and hence it is that bad habits are difficult to cure. Hence that, like wounds, they can more readily be cured in youth

---

* Medical Critic and Psychological Journal, July, 1862.

† Bird on " Drink Craving."

than in old age—in youth, when the body and mind are plastic and pliable, than in old age, when it is rigid and unbending to all influences except decay.''*

* Bird's Physiological Essays.

